# Glancing Angle Deposition Effect on Structure and Light-Induced Wettability of RF-Sputtered TiO_2_ Thin Films

**DOI:** 10.3390/mi9080389

**Published:** 2018-08-04

**Authors:** Vasiliki E. Vrakatseli, Alexandros N. Kalarakis, Angelos G. Kalampounias, Eleftherios K. Amanatides, Dimitrios S. Mataras

**Affiliations:** 1 Department of Chemical Engineering, University of Patras, GR-26504 Patras, Greece; vasvrakatseli@gmail.com (V.E.V.); alexandros_kalarakis@yahoo.gr (A.N.K.); dim@chemeng.upatras.gr (D.S.M.); 2 Department of Mechanical Engineering, Technological-Educational Institute of Western Greece, GR-26334 Patras, Greece; 3 Department of Chemistry, University of Ioannina, GR-45110 Ioannina, Greece; angelos@chemeng.upatras.gr; 4 Institute of Chemical Engineering Sciences/FORTH, P.O. Box 1414, GR-26504 Patras, Greece

**Keywords:** sputtering, glancing angle, TiO_2_, superhydrophilic, hydrophilic, anatase, rutile, GLAD, porous titania

## Abstract

Crystalline TiO_2_ films were prepared on unheated glass substrates by radio frequency (RF) reactive magnetron sputtering at normal angle of incidence (*a* = 0°) and at glancing angle (*a* = 87°). The effect of the glancing angle on the structure, microstructure, and wetting properties of the films was investigated. The inclination of the substrate led to phase transformation of the deposited films from rutile to either rutile/anatase or anatase, depending on the working pressure. Extreme shadowing at 87° results in a remarkable increase of the films’ porosity and surface roughness. The mechanism of the glancing-angle-induced crystalline phase formation is thoroughly discussed based on the thermodynamic, kinetic, and geometrical aspects of the nucleation and is related with the microstructural changes. Both crystalline phase and microstructure significantly affect the wetting properties of the TiO_2_ films. Glancing-angle-deposited anatase TiO_2_ exhibits a high degree of porosity and roughness, a high rate of UV-induced wettability conversion, and a long-term highly hydrophilic nature in dark. Therefore, anatase TiO_2_ is potentially a good candidate for applications as dye-sensitized solar cells (DSSC)/perovskite solar cells, microfluidic devices, and self-cleaning surfaces prepared on thermosensitive substrates.

## 1. Introduction

Titanium dioxide (ΤiO_2_) is a wide-band-gap semiconductor with unique properties for numerous technological applications. Optimization of the TiO_2_ thin films requires proper control of the microstructure along with the crystallinity and crystalline phase composition of the material. Three main TiO_2_ crystalline phases have been reported for TiO_2_: rutile, which is thermodynamically stable; anatase (metastable); and brookite (unstable). Among them, anatase is considered to be superior over rutile for applications as gas sensors and dye-sensitized solar cells (DSSC)/perovskite solar cells where improved photocatalytic functionality, charge separation, and transport efficiency are requisite [1,2]. Microstructure control towards high surface area and roughness has a pivotal role in the wetting properties (i.e., fast photoinduced reversible wettability or prolonged superhydrophilicity) of the anatase TiO_2_ films for microfluidic devices and self-cleaning surfaces applications [3,4,5]. At the same time, low-temperature deposition of TiO_2_ thin films with tailored properties attracts considerable interest since it can extend its use on thermosensitive substrates.

Liquid-phase deposition methods (sol-gel, hydrothermal, etc.) are simple and economic for the deposition of high-surface-area porous TiO_2_ films, but they suffer from mechanical instabilities and residual impurities, low crystallinity, and poorer wetting properties, unless the films are post annealed at high temperature [6,7]. RF/DC magnetron sputtering is a versatile vapor deposition method exhibiting remarkable results in the low-temperature deposition of crystalline thin films and is also suitable for large-area depositions [8,9,10,11]. In the absence of substrate heating, a crystalline structure is obtained due to the electron and ion bombardment during deposition, which provides a sufficient amount of energy for crystallization but does not severely raise the temperature of the bulk substrate. Both rutile and anatase TiO_2_ thin films have been deposited on unheated substrates by tuning different magnetron sputtering process parameters [8,9,10,11]. Room-temperature deposition of the anatase phase has been accomplished at elevated total and/or oxygen partial pressure [10,12,13,14,15], at larger target-to-substrate distances [11], or on a crystalline substrate [16]. Okimura et al. prepared all three amorphous, rutile, and anatase TiO_2_ at low temperature by altering the radial position of the substrate and pressure [14]. 

Glancing angle (or oblique angle) deposition (GLAD) refers to the configuration where the substrate is inclined with respect to the target. The particles ejected from the material source arrive on the surface of the substrate at an oblique angle and, due to the self-shadowing effect, nanostructured films with high surface area and porosity can be prepared [17]. Nevertheless, deposition of both crystalline and porous nanostructured TiO_2_ on unheated substrates by GLAD magnetron sputtering is rare compared to the normal incidence angle configuration. Mild substrate heating [1] or post annealing of the nanocolumnar films [6,18] usually follows GLAD in order to realize anatase films. RF magnetron-sputtered anatase TiO_2_ has been successfully deposited by Pihosh et al. on unheated substrates in both normal and GLAD configuration by working on a different pressure regime for each one [19]. However, the specific role of the GLAD geometry in crystallization and selective crystalline phase formation has not been reported yet. 

In this work, we report the GLAD-induced formation of anatase and mixed rutile/anatase over rutile phase on unheated substrates. The same deposition parameters are implemented for normal incidence or GLAD at two different pressures. Both microstructure and crystalline phase transition take place due to modifying the deposition geometry at an extreme 87° glancing angle. The GLAD-induced selective crystalline phase formation is examined, after taking into consideration the nuclei spatial distribution, the preferred shape mode of condensation of the as-grown TiO_2_, and the mechanism of formation of rutile phase over anatase. Furthermore, the TiO_2_ films deposited at both configurations are evaluated in terms of their wetting properties, the light-induced wettability efficiency, the reversible process in dark, and the stability of their performance. Glancing angle deposition significantly affects the wetting behavior of the films due to the change in their microstructure and crystalline phase. 

## 2. Materials and Methods

### 2.1. RF Reactive Magnetron Sputtering Depositions

The TiO_2_ films were prepared by the reactive RF (13.56 MHz) magnetron sputtering deposition technique on unheated glass substrates. The 3 cm × 2 cm glass substrates were cleaned in ultrasonic bath by hot acetone for 10 min, then rinsed in cold methanol and thoroughly dried by N_2_ gas. A 2 inch diameter Ti disc of 99.99% purity installed on the balanced magnetrons cathode (ST-20, AJA Int.) was used as the sputtered material. High-purity (99.999%) argon and oxygen were used as the sputtering and reactive gas, respectively. The gas mixture composition and the total working pressure were regulated by the relative Ar/O_2_ mass flow using different controllers (MKS, Andover, MA, USA). The RF power input was kept constant at 300 W and the oxygen molar ratio in the mixture with argon was set at 0.50. Before each deposition the chamber was evacuated to 4 × 10^−7^ Torr. The Ti target was cleaned by pre-sputtering with Ar for 5 min. 

Depositions of the F-coded samples were carried out at the normal a = 0° substrate angle and those of the G-coded samples at the extreme 87° glancing angle of the substrate with respect to the cathode axis. For each configuration the total working pressure was 1 mTorr (F_1, G_1) or 2.2 mTorr (F_2.2, G_2.2). The target to the substrate’s center distance was 10 cm, and 5 cm for the G5_2.2 coded sample. The thickness of all TiO_2_ films was at the same magnitude (~220 ± 20 nm), as the deposition time was properly adjusted according to preliminary growth rate experiments. No bias, rotation, or heating was applied to the substrate. The self-heating temperature of the substrate due to plasma sputtering was <150 °C for all the depositions, recorded by thermocouple attached to the side surface of the glass. The deposition conditions are summarized in Table 1.

### 2.2. TiO_2_ Thin Films Characterization

The TiO_2_ film thickness was measured by contact profilometry (DektakXt). The crystalline phase was identified by confocal UV–vis micro-Raman spectroscopy (Horiba Jobin-Yvon LabRam HR800, Horiba, Kyoto, Japan) at 441.6 nm excitation line of the HeCd laser (Kimmon Electric Co., Itabashi-Ku, Japan). In order to avoid laser-induced crystallization of the sample, the spectra were obtained using a fixed 0.07 mW laser power on the specimen. Short accumulation times were sufficient to achieve a high signal-to-noise ratio for all recorded Raman spectra. Microscopy images were obtained by Field Emission Scanning Electron Microscopy (FE−SEM, FEIInspectTM F50, FEI Company, Hillsboro, OR, USA) and the outermost surface morphology was acquired by Atomic Force Microscopy (Dimension Fast Scan, Bruker Corporation, Billerica, MA, USA). UV–vis transmittance spectra of TiO_2_ films were recorded using a Lambda900 UV–vis–nIR spectrophotometer on the single-beam mode at the range 1000–300 nm. The optical constants were calculated using the Pointwise Unconstrained Minimization Approach (PUMA) from the recorded transmission spectra [20] with maximum fitting error of the order of 5 × 10^−3^ [20]. PUMA is a nonlinear programming method for the estimation of the functions *n*(*λ*) (refractive index) and the attenuation coefficient *κ*(*λ*) of a thin film on a thick transparent substrate by using the measured transmittance data. The estimation of the unknowns *n*(*λ*) and *κ*(*λ*) derives from the continuous least squares solution of

(1) $$Minimize\;\int_{\lambda_{min}}^{\lambda_{max}}|Τ\left(\lambda ,s\left(\lambda \right),d,n\left(\lambda \right),\kappa \left(\lambda \right)\right)-{Τ}^{measured}\left(\lambda \right){|}^{2}\;d\lambda$$

The wetting properties of the RF-sputtered TiO_2_ films were evaluated by static contact angle goniometry (Kruss DSA-100S) [7]. All characterizations were performed using the same samples. 

## 3. Results and Discussion

### 3.1. Effect of the Glancing Angle Deposition on TiO_2_ Structure and Morphology

**Figure 1:**
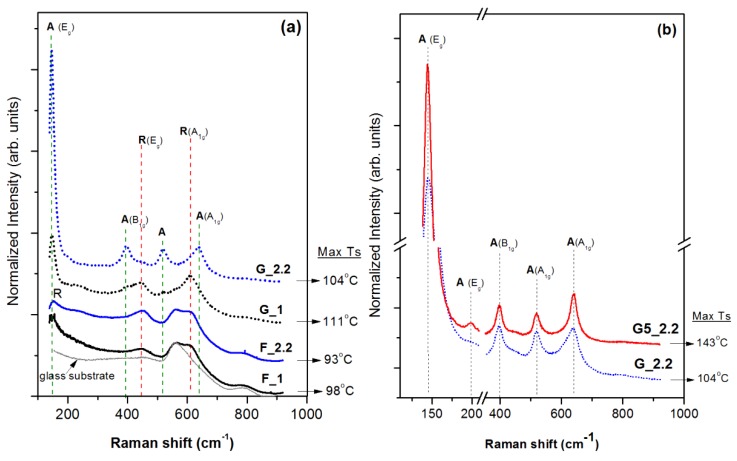
(**a**) Raman spectra of RF-sputtered TiO_2_ films deposited at 1 mTorr and 2.2 mTorr total gas pressure on 0°-inclined substrate (F_1, F_2.2) and on 87° glancing angle deposition (G_1, G_2.2) with regard to the target. (**b**) Raman spectra of 87° glancing-angle-deposited TiO_2_ films at 2.2 mTorr and 10 cm (G_2.2) and 5 cm (G5_2.2) substrate-to-target distance. The maximum substrate temperature is also shown here (as recorded at the side surface of the glass substrate).

Figure 1a shows the recorded Raman spectra of the RF-sputtered TiO_2_ films. The crystalline phase of the films is identified by the peak positions of the Raman active vibrational modes of rutile and anatase single-crystal phases. The reported active modes of vibrations for the single-crystal anatase TiO_2_ in the Raman region are 3Eg (144, 198 and 639 cm^−1^), B1g (399 cm^−1^), and A1g/B1g (516 cm^−1^), while the rutile phase exhibits five active modes of vibration: B1g (145 and 243 cm^−1^), Eg (447 cm^−1^), A1g (612 cm^−1^), and B2g (826 cm^−1^) [21].

The F_1 and F_2.2 sample spectra are quite similar, exhibiting peaks at ~614 cm^−1^, 451 cm^−1^, and at 150 cm^−1^, which correspond to the A1g, Eg, and B1g vibrational modes of rutile. The higher wavenumber peak shift relative to the reported values may reflect residual stresses on the 0°-angle-deposited TiO_2_ films [22]. The peak at 562 cm^−1^, found in both F_1 and F_2.2 spectra, derives from the apparent contribution of the glass spectrum, implying the amorphous content of the films. Overall, the change in pressure from 1 to 2.2 mTorr does not affect the microstructure of the films deposited at 0°.

On the other hand, the change of the substrate inclination angle from 0° to 87° has a significant impact on the crystalline phase of TiO_2_ films deposited at both 1 mTorr and 2.2 mTorr. A mixed rutile/anatase phase is recognized at the G_1 spectrum. The peaks at 609 cm^−1^ and 447 cm^−1^ correspond to the rutile phase, but anatase is also identified by the characteristic Eg vibration mode at 143 cm^−1^ and the appearance of the weaker peaks at 398 and 518 cm^−1^. However, the structure is drastically changed for the G_2.2 film (2.2 mTorr, 87°) where concrete anatase crystalline phase is identified by the well-defined Raman features associated with the anatase vibrational modes. Thus, the Raman show the following: (a) The increase of the sputtering pressure from 1 mTorr to 2.2 mTorr influences only the glancing angle depositions, resulting in phase change from rutile/anatase (G_1) to the predomination of anatase in the G_2.2 film, and (b) The increase of glancing angle from 0° to 87° induces phase change at both pressures but the effect is much stronger at 2.2 mTorr where transformation from rutile (F2_2) to anatase (G2_2) was observed.

It is well known that the bulk anatase crystal is a metastable TiO_2_ phase, whereas the bulk rutile phase is thermodynamically stable. Nevertheless, at the early growth stage, anatase formation is favored from a structural point of view due to the facile arrangement of the short-range order TiO_6_ octahedra into long-range ordered anatase phase [23]. Furthermore, the rutile surface free energy is higher than that of anatase (1.9 J/m^2^ over 1.3 J/m^2^, respectively) and, thus, the thermodynamic stability turns out to depend on the volume-to-surface-area ratio of the particles, favoring the formation of the anatase phase for particles up to ~14 nm as reported by Zhang and Banfield [24,25]. The rutile phase is formed by the surface and interface nucleation of nanosized anatase [26]. Therefore, the formation of rutile islands at the early stages of the deposition is anatase size dependent [24,25,26,27,28]. Once the anatase nanoparticles transition to rutile initiates, the exothermic phase transformation speeds up the process itself, causing migration towards the exterior of the new particle and the irreversible formation of rutile particles larger than twice the size of the initial anatase nanoparticles [29]. However, if the nucleation density at the initial stages of the film growth is sufficiently low, anatase grows large enough with a higher rate than the rutile nuclei, eventually suppressing the formation of rutile [30]. Therefore, inhibition of the formation of the rutile phase could be achieved either by controlling the early stage nucleation density, or the size and shape of the nucleation islands by keeping a low volume-to-surface-area ratio, or both.

Based on the discussion above, parameters that affect nucleation density and morphology of nucleation islands can determine the phase of the deposited films. Plasma-induced surface heating and the film growth rate can have such an effect; thus, their values are summarized in Table 1 for all films. The substrate temperature that is reached in each deposition is about the same, so it cannot be responsible for the observed phase changes. However, the glancing-angle-deposited TiO_2_ films suffer from a severe decrease of the growth rate (Table 1). For instance, the deposition rate of the F_2.2 sample is about 3.7 nm/min, but the increase of the glancing angle from 0° to 87° (G_2.2) leads to a severe drop of the film growth rate to 0.75 nm/min, while all other process parameters remain the same. In order to investigate if the film growth rate is the main factor that determines the observed phase changes, one more deposition was performed at the conditions of film G_2.2 but at a shorter target-to-substrate distance (*d_t_*_−*s*_ = 5 cm, G5_2.2). This change induces a significant increase of the film growth rate from 0.75 nm/min to 2.3 nm/min, i.e., a rate that is comparable to that of the 0° deposition process. Figure 1b shows the Raman spectra of the G_2.2 film and G5_2.2 TiO_2_, both presenting the characteristic peaks of anatase. Therefore, the change of the crystalline phase from the nanorutile F_2.2 sample to the anatase G_2.2 cannot be attributed to the reduction of the deposition rate. The geometry of the deposition, the species angle of incidence, and the self-shadowing effect need to be further discussed to understand the observed phase transitions.

The spatial arrangement of the sputtering particles on a room-temperature substrate will be controlled by the number and the set of directions of incidence, due to the limited adatom mobility [31]. As the first particles arrive on the cold substrate, some of them bind on the energetically favored substrate sites, i.e., crystalline sites or on substrate point defects, while the rest will probably reevaporate [32]. The adatoms or clusters arriving next will preferentially attach to each other rather than to the cold substrate, enabling the initiation of growth of three-dimensional islands, according to the Volmer–Weber thin film growth model [32]. At the normal deposition of 0° (F samples), the sputtered particles (neutrals or charged clusters) will be distributed on the cold substrate quite homogeneously, since they approach with a wide distribution of incident angles depending on substrate-to-target distance, the sputtering pressure, and the relative substrate–target area. Τhe 0°-inclined substrate is “sprayed” by the sputtering particles and the nucleation islands probably develop in a comparable rate along the *x*–*y* surface or the *z* axis. Planar growth of the islands will additionally be facilitated at the low-pressure deposition F_1 where the incident particles possess a higher kinetic energy, while sufficient electron and ion bombardment of the substrate can also enhance adatom diffusion by surface heating of the substrate. In this case, there is a great probability for the initial nanosized anatase to convert to rutile; this is what we observe in our experiments for the 0° deposited films (F_1, F_2.2).

Conversely, on the 87°-inclined substrate, the angle of the arrival flux is narrowed and the total number of the incident particles per unit area of the substrate is decreased, resulting in low deposition rates. Due to the reduced number of incoming species per substrate site available for nucleation, the sticking probability will be lower compared to that for the normal incidence angle deposition. Consequently, the species that stick to the substrate will provoke early shadowing effects and, as a result, the nucleation islands will be more sparsely dispersed. The *z*-axis growth will be enhanced over planar growth of the islands due to shadowing effect of the already formed islands. The ratio of volume to surface area of the nucleation island will be lower in this case, favoring the formation of anatase over rutile; this is confirmed in our experiments for the 87° deposited films and especially in the high-pressure case (G_2.2, G5_2.2). 

**Figure 2:**
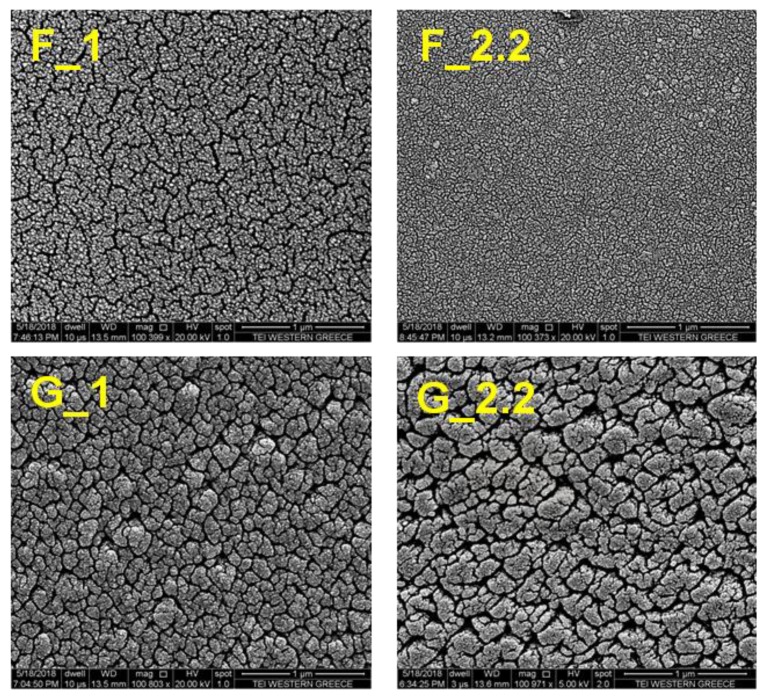
Top-view SEM micrographs of the TiO_2_ films. F_1: *a* = 0°, *P* = 1 mTorr, F_2.2: *a* = 0°, *P* = 2.2 mTorr, G_1: *a* = 87°, *P* = 1 mTorr, G_2.2: *a* = 87°, *P* = 2.2 mTorr. The scale bar is 1 μm for all images.

According to the above discussion, the deposition of rutile or anatase is determined from planar over *z*-axis growth of islands; thus, films of different crystalline phase are expected to present strong differences in their morphology. Figure 2 shows the top-view SEM micrographs of the TiO_2_ films grown at deposition angles of 0° and 87°. The structure zone model [31] describes the expected microstructure for thin films deposited by physical vapor deposition at the normal angle of incidence flux. The resulting morphology is considered to be a function of the homologous temperature *T_h_* (i.e., the ratio of substrate temperature *Ts* to the melting temperature of the deposited material *Tm*) and of the process pressure [31]. The films deposited at normal incident flux F_1 and F_2.2 are in agreement with the Structure Zone 1 model, which predicts loosely packed films consisting of tapered units defined by voided boundaries, as a result of the limited adatom mobility on the low-temperature substrate [33]. A larger scale void network is observed for F_1 compared to the F_2.2 film mainly due to the increase of the energetic particles flux, the increase of particles kinetic energy, and the enhancement of film intrinsic tensile stress at lower pressure [34]. 

GLAD samples are completely different due to the shadowing effect, exhibiting large-sized grains and increased porosity as well as surface roughness. Higher GLAD angles can result in the increase of the intercolumnar distance diameter, as observed for TiO_2_ films deposited by electron beam evaporation [35]. Here, we observe the same effect in G_2.2 compared to the G_1 film just by the small increase in the sputtering pressure. At higher pressures, the sputtering particles experience more collisions as they travel to the substrate and they finally arrive with a decreased momentum; thus, their transportation to voids or to the smaller columns will be inhibited [36]. Eventually, more of the smaller columns will be overshadowed, leaving voids as the film grows. However, the angular distribution of the arrival flux will be wider at the higher-pressure conditions and we expect that the column diameter size will increase with deposition time. This is also observed in our experiments as the film deposited at higher pressure (G_2.2) presents larger voids and grains compared to G_1. 

**Figure 3:**
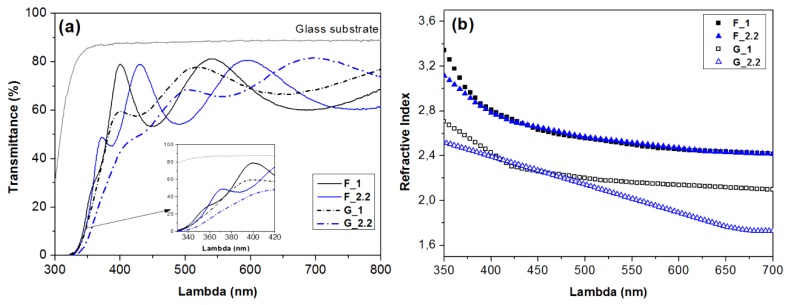
(**a**) UV–vis transmittance spectra of the 0°-substrate-angle-deposited films (solid lines) and 87° glancing-angle-deposited films (dashed lines) and (**b**) the corresponding refractive indices, estimated using the Pointwise Unconstrained Minimization Approach (PUMA) computational method [20].

Differences in the films’ morphology and crystalline phase will affect the optical properties of the materials. The recorded transmittance spectra of the RF-sputtered TiO_2_ films on the glass substrate are shown in Figure 3a. The maximum transmittance of the F_1 and F_2.2 samples is 80% and those of the G_1 and G_2.2 films are 79% and 82%, respectively. The UV cut-off wavelength of the G_2.2 anatase film is shifted to a longer wavelength, implying a smaller optical band gap compared to the rest. The observed difference in the number and position of the interference fringes between the F_1 and F_2.2 samples derives from the small variation of the film thickness (240 nm vs 210 nm). Although the thickness magnitude is practically the same for the F and G films at ~220 ± 20 nm, the constructive and destructive interference fringes of the G samples are overall less intense, indicating a smaller effective refractive index. The refractive index was calculated using the Pointwise Unconstrained Minimization Approach (PUMA) [20] with a fitting error on the order of 10^−4^ to 10^−3^ and is presented for all films as a function of the wavelength in Figure 3b. 

Subsequently, the porosity was estimated using the formula provided by Yoldas [37]:

(2) $$\mathrm{Porosity}\%\;=\;\left[1\;-\left(\frac{n_{f}^{2}\;-\;1}{n_{b}^{2}\;-1}\right)\right]\;\times \;100\text{\%}$$

where *n_f_* is the refractive index of the deposited TiO_2_ film and *n_b_* is the reported refractive index of the bulk single-crystal TiO_2_. The porosity of the F samples was calculated with respect to rutile’s *n_b_* (2.7 at 600 nm) and that of the G samples with respect to the anatase *n_b_* (2.534 at 600 nm). The calculated values of % porosity are presented in Table 1 and, as we can observe, the GLAD TiO_2_ films exhibit much higher porosity, which is in agreement with the SEM images and the discussion for the mechanism of anatase and rutile growth.

**Figure 4:**
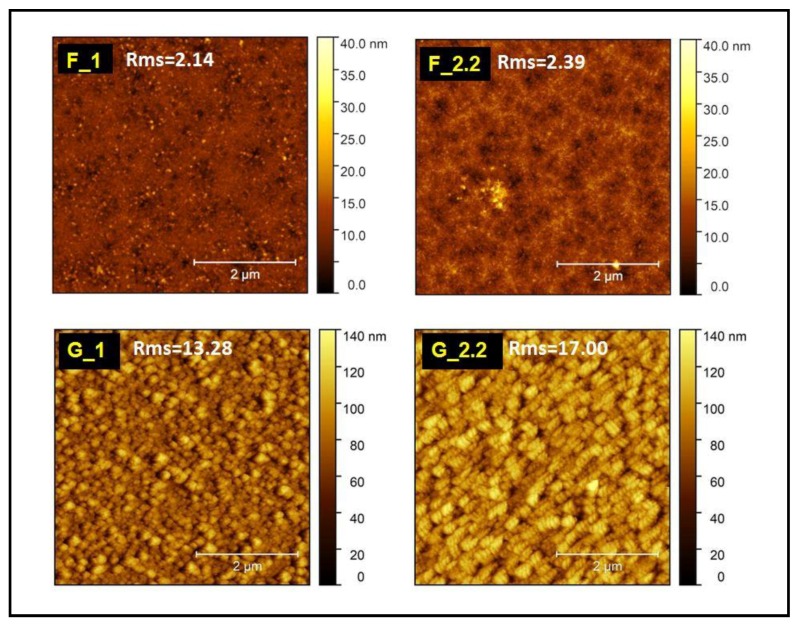
2D AFM images and the average RMS surface roughness of (**a**) F_1, (**b**) F_2.2, (*z* scale: 0–40 nm) (**c**) G_1 and (**d**) G_2.2 (*z* scale: 0–140 nm); films all shown in an *x*–*y* scale of 5 μm × 5 μm.

Along with the porosity and phase change, the surface roughness of the TiO_2_ thin films is significantly changed for the glancing angle depositions. Figure 4 presents the 2-dimensional atomic force microscopy images of the F and GLAD TiO_2_ thin films together with their corresponding root mean square (RMS) roughness. The TiO_2_ films deposited at the normal 0° substrate angle are quite smooth with average surface roughness of 2.14 nm and 2.39 nm for F_1 and F_2.2 films. The pressure increase for the 0° depositions has a negligible effect on the surface roughness. On the other hand, the films deposited at the 87° glancing angle exhibited higher surface roughness. The average RMS values calculated for G_1 and G_2.2 are 13.28 and 17.00 nm, respectively. The surface roughness has a key role and strongly affects the wetting properties of the deposited TiO_2_ films, which we will examine next. 

### 3.2. Effect of Glancing Angle Deposition on Wetting Properties of the TiO_2_ Thin Films

All the RF-sputtered thin films were superhydrophilic (<10°) immediately after the deposition. The change in the wettability of the TiO_2_ films was recorded via water contact angle measurements for two months of storage in dark ambient conditions. The F_1 and F_2.2 samples become hydrophobic within 25 days with contact angle at 96.5°. The GLAD-deposited films exhibited high hydrophilicity for an extended period. The G_1 contact angle increased much slower than did that of the flat-deposited samples and eventually turned hydrophobic after almost 2 months. The as-deposited G_2.2 film exhibited stable hydrophilic character with contact angle <20° for more than 20 days and stabilized at values of about 45° after two months of storage in dark ambient conditions. 

**Figure 5:**
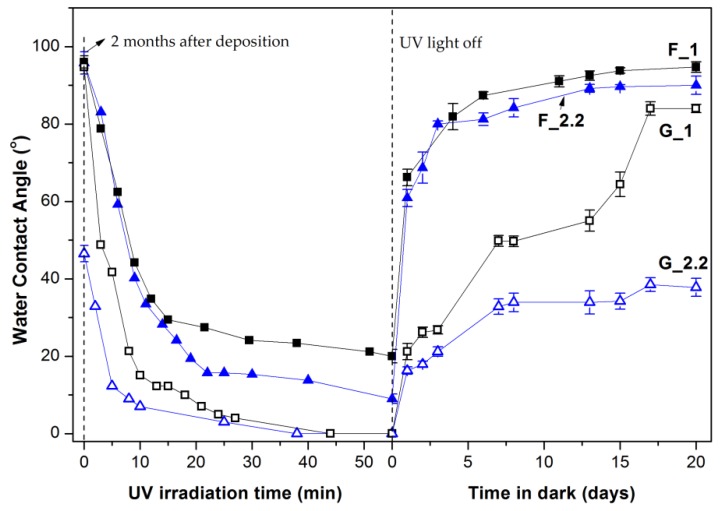
Variation of the water contact angle of the TiO_2_ films with UV irradiation (8 mW/cm^2^) time and with storage time of the samples in dark and ambient conditions.

**Figure 6:**
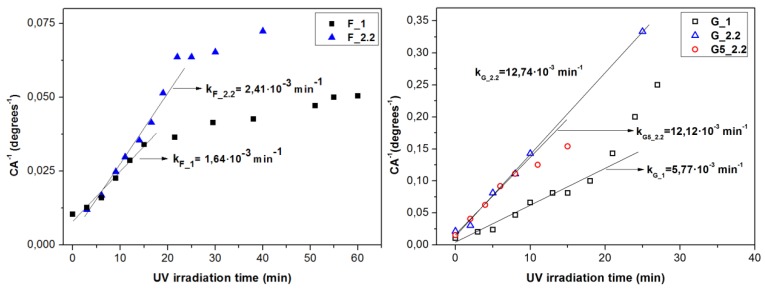
The reciprocal of the contact angle versus the UV irradiation time and the slope of the linear region representing the UV hydrophilic conversion rate coefficient. Left: F-samples deposited at *a* = 0°; Right: G samples deposited at *a* = 87°.

Figure 5 shows the change of the measured contact angle of the F and G samples under UV irradiation (8 mW/cm^2^) and in dark. The prevailing mechanism of the UV-induced hydrophilicity of TiO_2_ suggests that the photogenerated electron-holes cause the induction of surface oxygen vacancies which consecutively act as energetically favored sites for the dissociative adsorption of water [38,39,40]. In contrast to the normally deposited samples, all GLAD samples (G5_2.2 not shown here) reach the complete wetting state (0°) during the applied UV irradiation time. In order to evaluate the UV-induced hydrophilic conversion independent of the initial contact angle, we have adopted the method proposed by Sakai et al. [39]. The reciprocal contact angle is plotted versus the UV illumination time and the hydrophilic conversion rate is evaluated from the slope of the linear region as in Figure 6. The hydrophilic conversion rate coefficient *k* increases as follows: F_1 < F_2.2 << G_1 << G5_2.2 ≅ G_2.2. The correlation coefficients of the linear fittings are 0.968, 0.982, 0.979, 0.999, and 0.999, respectively.

The overall rate of the contact angle recovery in dark follows the opposite scheme: G5_2.2 ≅ G_2.2 < G_1 << F_2.2 < F_1. Apparently, the reversible process in dark is remarkably slow for the GLAD TiO_2_ films, while for the 0°-angle-deposited films it is similar to that of amorphous TiO_2_ [5]. Ιn dark conditions, the contact angle increases due to the desorption of physisorbed water and the substitution of the metastable chemisorbed hydroxylic moieties by the ambient oxygen [5,38,39]. Nanostructured anatase has been found to possess a higher amount of intrinsic oxygen vacancies [5] and, therefore, a higher amount of chemisorbed hydroxyls is expected on the anatase surfaces. Enhanced hydroxylation combined with higher surface roughness and porosity would cause the extended retardation of the contact angle recovery in dark for the rutile–anatase G_1 and anatase G_2.2 TiO_2_ films.

Furthermore, the GLAD sample contact angle recovery proceeds in different rate steps within the same period of time in dark. The different behavior of the nanostuctured GLAD TiO_2_ films in dark can be associated with the more complex microstructure, the higher surface area, and the wider surface pore distribution. The physisorbed hydroxyls and those chemisorbed on the upper surface grains which are in full contact with the ambient air will disengage first. Next, the dehydroxylation of the surface will proceed according to the pore density, shape, and distribution, depending on the contact area of the ambient air and the specific pore feature.

**Figure 7:**
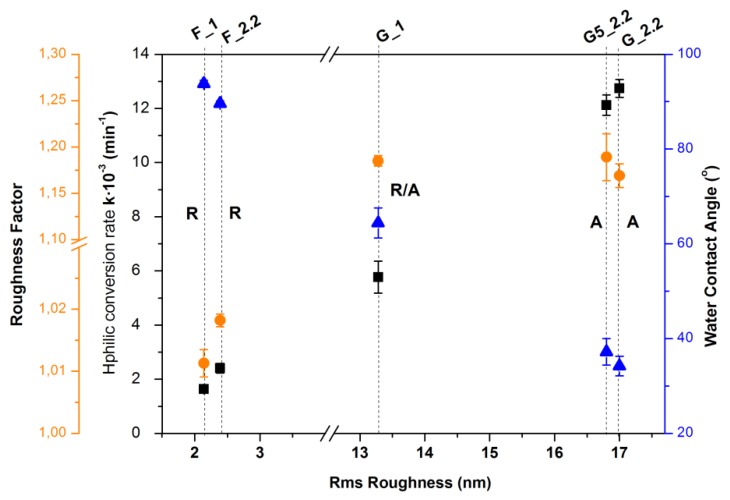
Correlation of the TiO_2_ films wetting properties with the average RMS roughness and the corresponding roughness factor. *R* and *A* symbols indicate the crystalline phase. Black symbols: hydrophilic conversion rate constant (min^−1^). Blue symbols: measured contact angle after 15 days in dark ambient conditions. Orange symbols: surface roughness factor defined as real surface area/apparent area.

The correlation of the wetting characteristics of the TiO_2_ films with the RMS roughness is shown in Figure 7 and their corresponding values in Table 2. The microstructure characteristics will have an impact on the absolute value of the water contact angle on the surface, as has been extensively described by several models [5,41,42]. The effect of the roughness factor is stronger as the contact angle value decreases; thus, a higher roughness factor induces faster hydrophilic conversion of the surface. The roughness factors of F and G TiO_2_ films were calculated from the 5 μm × 5 μm AFM images according to the dynamic scale theory [5,43]. 

For the 0°-angle-deposited amorphous rutile TiO_2_ films and the rutile/anatase G_1 film, the increase of the hydrophilic conversion rate is directly correlated to the increase of the surface roughness and the roughness factor. However, the rate of the anatase films is doubled compared to the mixed-phase rutile anatase G_1, irrespective of the initial contact angle and despite the fact that the roughness factor is actually the same or even reduced. It is worth noticing that the reciprocal contact angle hydrophilic conversion rate does not depend on the initial contact angle and therefore reflects the rate of the hydroxylation of the surface instead of its absolute wettability [39]. The anatase TiO_2_ films also exhibit a significantly slow contact angle conversion in dark as can be evaluated by the contact angle after 15 days in dark, when the F samples have already reached their initial hydrophobic wetting state. 

Therefore, the wetting behavior of the GLAD anatase samples cannot be exclusively attributed to the glancing-angle-induced microstructure. It has been proved that the anatase phase contains inherent oxygen defects [44], and photoluminesence studies on GLAD nanocolumnar TiO_2_ films also revealed that the improved photocatalytic activity of anatase is related with the oxygen defects present in the crystal [6].The intrinsic-oxygen-defect-rich anatase [44] is provided with permanent sites which thermodynamically favor the association of ambient hydroxyls. Combined with the GLAD-induced microstructural characteristics (high surface roughness and porosity), faster hydroxylation of the surface and stable high hydrophilicity is achieved. 

**Figure 8:**
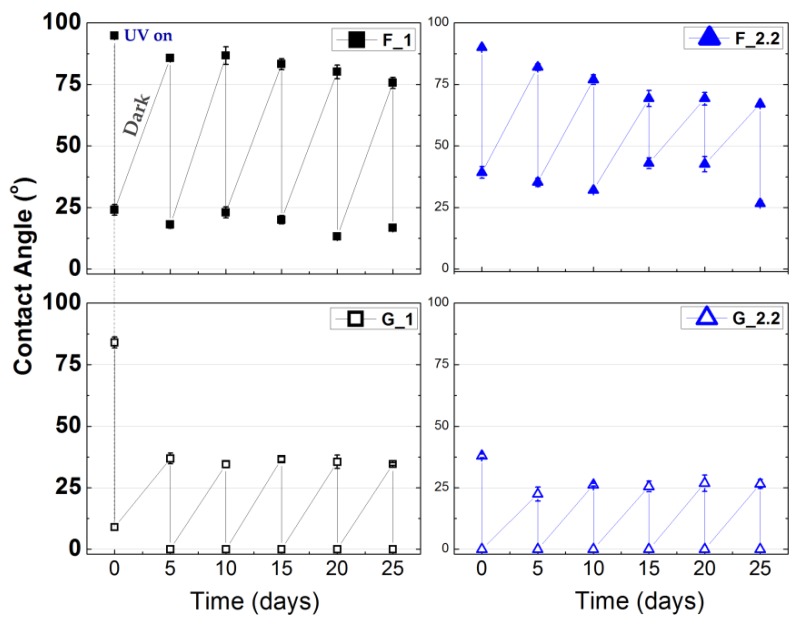
Water contact angle changes of RF-sputtered TiO_2_ thin films in five cycles of 30 min UV illumination and consecutive storage in dark and ambient conditions for 5 days.

Finally, for the evaluation of the long-term performance, the TiO_2_ films were also subjected to fixed time, recursive UV on/off cycles for one month. Figure 8 shows the measured contact angles of the samples after 30 min of UV illumination and after 5 days in dark ambient conditions. The 0°-angle-deposited TiO_2_ films’ UV sensitivity deteriorates with time, which may be an effect of the tensile stress in the F films [45]. The GLAD samples present a steady change from highly hydrophilic to superhydrophilic from cycle to cycle. Therefore, the contact angle deviation can be effectively controlled by the application of short-time UV illumination so that a permanently stable superhydrophilic anatase TiO_2_ film is obtained at low substrate temperature.

## 4. Conclusions

TiO_2_ thin films were prepared on unheated glass substrates by RF reactive magnetron sputtering at normal 0° incidence angle and at the extreme 87° glancing angle. The depositions were carried out under a low-pressure regime (1 mTorr, 2.2 mTorr) while all the other deposition parameters maintained constant. Crystalline rutile, mixed rutile/anatase, and anatase TiO_2_ films were obtained as confirmed by micro-Raman studies of the as-deposited TiO_2_ films. The role of the geometry of the deposition was found to be crucial for the selective formation of the crystalline phase combined with significant microstructural changes of the GLAD TiO_2_ films. Rutile TiO_2_ films were deposited at the normal 0° incidence angle. The change of glancing angle from 0° to 87° resulted in mixed rutile/anatase phase for the lower-pressure deposition (1 mTorr) and in anatase phase for the higher pressure (2.2 mTorr). Although the GLAD films exhibit low deposition rates (<1 nm/min), we have shown for the anatase film that the deposition rate can be significantly enhanced by reducing the substrate-to-target distance while the anatase phase is retained.

Due to the shadowing effect, glancing-angle-deposited films exhibited increased porosity up to 53% as well as increased surface roughness. Based on the thermodynamic aspects of the existing phase transition models, selective crystalline phase formation was attributed to the spatial and geometrical characteristics of the nucleation and growth and to the pressure-modulated energetic profile of the deposition. Control of the wetting properties of the deposited TiO_2_ films was assessed as a matter of both microstructure and the crystalline phase present. The rutile TiO_2_ films (*a* = 0°) with low roughness and porosity were relatively hydrophobic and showed limited UV-induced functionality and stability compared to the corresponding GLAD films. The glancing-angle-deposited anatase films exhibited a higher rate of UV-induced superhydrophilicity and long-term hydrophilic surface in dark compared to the mixed rutile/anatase, despite the smaller roughness factor. RF reactive magnetron deposition at the extreme glancing angle of 87° appears to be an effective method for the low-temperature preparation of anatase TiO_2_ with high porosity, high surface roughness, and superior wetting properties.

## Figures and Tables

**Table 1:** Deposition parameters, sample nomenclature, maximum deposition temperature, film thickness, porosity, and the corresponding crystalline phase.

Substrate Configuration	Sample	Target-to-Substrate Distance (cm)	Pressure (mTorr)	Dep. Rate (nm/min)	SbstrTmax (°C)	Film Thickness (±5 nm)	Crystalline Phase	Porosity (%)
Normal *a* = 0°	F_1	10	1	3.7	98	240	Rutile	19
	F_2.2	10	2.2	3.67	93	210	Rutile	20
GLAD *a* = 87°	G_1	10	1	0.65	111	240	Rutile/Anatase	34
	G_2.2	10	2.2	0.75	104	225	Anatase	52.6
	G5_2.2	5	2.2	2.24	143	245	Anatase	n.a.

**Table 2:** The TiO_2_ thin films surface properties (RMS roughness and roughness factor), hydrophilic conversion rate, water contact angle (WCA) and their corresponding crystalline phase.

Sample	Crystalline Phase	RMS Roughness (nm)	Roughness Factor	Hydrophilic Conversion Rate *k* × 10^−3^ (min^−1^)	WCA after 15 Days in Dark (Degrees)
F_1	Rutile	2.14	1.0113	1.64	93.8
F_2.2	Rutile	2.39	1.0182	2.41	89.6
G_1	Rutile/Anatase	13.28	1.185	5.77	64.4
G5_2.2	Anatase	16.81	1.189	12.12	37.2
G_2.2	Anatase	17.00	1.169	12.74	34.4
